# Predictors of Enrolment in the National Health Insurance Scheme Among Women of Reproductive Age in Nigeria

**DOI:** 10.15171/ijhpm.2018.68

**Published:** 2018-08-06

**Authors:** Bolaji Samson Aregbeshola, Samina Mohsin Khan

**Affiliations:** ^1^Department of Community Health & Primary Care, College of Medicine, University of Lagos, Lagos, Nigeria.; ^2^Department of Public Health Sciences, Karolinska Institutet, Stockholm, Sweden.

**Keywords:** National Health Insurance, Enrolment, Women, Universal Health Coverage, Nigeria

## Abstract

**Background:** Despite the implementation of the National Health Insurance Scheme (NHIS) since 2005 in Nigeria, the level of health insurance coverage remains low. The study aims to examine the predictors of enrolment in the NHIS among women of reproductive age in Nigeria.

**Methods:** Secondary data from the 2013 Nigeria Demographic and Health Survey (NDHS) were utilized to examine factors influencing enrolment in the NHIS among women of reproductive age (n=38 948) in Nigeria. Demographic and socio-economic characteristics of women were determined using univariate, bivariate and multivariate analyses. Data analysis was performed using STATA version 12 software.

**Results:** We found that 97.9% of women were not covered by health insurance. Multivariate analysis indicated that factors such as age, education, geo-political zone, socio-economic status (SES), and employment status were significant predictors of enrolment in the NHIS among women of reproductive age.

**Conclusion:** This study concludes that health insurance coverage among women of reproductive age in Nigeria is very low. Additionally, demographic and socio-economic factors were associated with enrolment in the NHIS among women. Therefore, policy-makers need to establish a tax-based health financing mechanism targeted at women who are young, uneducated, from poorest households, unemployed and working in the informal sector of the economy. Extending health insurance coverage to women from poor households and those who work in the informal sector through a tax-financed non-contributory health insurance scheme would accelerate progress towards universal health coverage (UHC).

## Background

Expanding health insurance coverage to people in the informal sector of the economy is a major challenge in most low- and middle-income countries (LMICs). This is due to large informal sector populations in these countries. According to International Labour Office (ILO), over 60% of the world’s employed population are in the informal sector of the economy.^1^ Developing countries have about 90% of informal employment as a percentage of total employment.^1^ However, Africa has about 86% of informal employment as a percentage of total employment.^1^ Nigeria has over 90% of informal employment as a percentage of total employment.^1^ The informal sector comprises mostly of the poor and vulnerable groups who are unable to pay for healthcare or work in enterprises where it is difficult to collect insurance contributions.^2,3^

Health insurance has been shown to be an important health financing mechanism in improving access to healthcare services and providing financial risk protection.^4-12^ Furthermore, there is a global call for countries to move towards universal health coverage (UHC) through sustainable health financing.^13^ UHC has become an important target of the Sustainable Development Goals (SDGs).^14^ UHC aims to increase equity in access to quality healthcare services and reduce associated financial risk.^15^

According to the World Bank and World Health Organization (WHO), at least half of the world’s population still lacks access to essential health services.^16^ The 2018 World Health Statistics also revealed that the UHC service coverage index for Nigeria is 39%.^17^ Pre-payment mechanism has been advocated as the best option to ensure access to care based on need rather than ability to pay and protect households from associated financial risk.^13^ Despite the implementation of the National Health Insurance Scheme (NHIS) since 2005 in Nigeria, the level of health insurance coverage remains low.^18-20^ Therefore, there is a need to understand the factors responsible for the low level of enrolment in the NHIS.

Many studies have been conducted on the determinants of NHIS enrolment in Africa.^3,21-42^ Results from these previous studies revealed that factors such as age, education, place of residence, region of residence, marital status, ethnicity, employment status, household wealth, gender, household size and exposure to media were predictors of enrolment in the NHIS. However, there is limited evidence on the predictors of enrolment in the NHIS among women of reproductive age in Nigeria using a nationally representative household survey. Evidence suggests that gender plays an important role in NHIS enrolment.^28^ Women face different health risk and difficulties in accessing healthcare services.^34^ They also bear a greater burden of disease and have limited access to resources.^29,34^ The ILO revealed that a higher percentage of women are in informal employment than men.^1^ It is against this background that the study aims to examine factors associated with enrolment in the NHIS among women of reproductive age in Nigeria in order to inform policy decision-making towards addressing the problem of low enrolment in the NHIS. The study contributes to the literature in Africa and to better understanding of the correlates of enrolment in the NHIS among women of reproductive age in Nigeria.

## Overview of the National Health Insurance Scheme in Nigeria

After many attempts at having legislation on health insurance since the 1960s, the NHIS, although established in 1999, became operational in 2005 to ensure access to quality healthcare services, provide financial risk protection, reduce rising cost of healthcare services and ensure efficiency in healthcare.^43^ The NHIS law made enrolment in the scheme optional.^43^ NHIS membership is mandatory for workers in the formal sector while it is voluntary for those in the informal sector.^43^ Formal sector workers, a spouse and their dependents under the age of 18 years are automatically enrolled into the NHIS based on a 5% monthly contribution from worker’s basic salary and payment of 10% of worker’s basic salary by the employer.^43^ Informal sector workers are expected to voluntarily enrol by making annual premium payments which varies depending on the health insurance plan.^43^ States are not legally mandated to provide health insurance to the people^43^ but there is a current drive to decentralise social health insurance (SHI) scheme to the states. NHIS has been implemented through programmes such as Formal Sector Social Health Insurance Programme (FSSHIP), Mobile Health, Voluntary Contributors Social Health Insurance Programme (VCSHIP), Tertiary Institution Social Health Insurance Programme (TISHIP), Community Based Social Health Insurance Programme (CBSHIP), Public Primary Pupils Social Health Insurance Programme (PPPSHIP) and the Vulnerable Group Social Health Insurance Programme (VGSHIP) which aims to provide healthcare services for children under 5 years, pregnant women, prison inmates, disabled persons, retirees and the elderly.^43^ The NHIS target different population groups including women and those in the informal sector with the aim of working toward UHC.^44^ Over a decade since its implementation, evidence suggests that the NHIS has provided health insurance coverage to less than 5% of the Nigerian population.^18-20^ This implies that the NHIS has failed to reach all population groups especially the poor, vulnerable and informal sector populations. Consequently, the poor, vulnerable and informal sector populations have to pay out-of-pocket (OOP) for healthcare services. OOP payments continue to be a major source of financing healthcare in Nigeria.^45^ In addition, OOP payments have been regarded as an inequitable source of financing healthcare^46^ with catastrophic and impoverishing effects on individuals and households. Globally, over 800 million people spend at least 10% of their household budget paying for healthcare while about 100 million fall into extreme poverty due to OOP health payments.^16^ A study on the catastrophic and impoverishing effects of OOP health payment in Nigeria revealed that about 17% of households incurred catastrophic health payments at 10% threshold of total consumption expenditure while about 1.3 million Nigerians were being pushed below the poverty line due to OOP health payments.^47^ However, enrolment in health insurance increases the likelihood of using general healthcare.^5,48-50^ SHI programme has entrenched inequities in access to healthcare as only federal government workers and their dependents are provided with health insurance coverage.^18^ Furthermore, the better-offs are more likely to enrol in the NHIS than poor households. This could be due to poor household’s low earning capacity, inability to pay premium contributions and poor awareness. Community-based health insurance (CBHI) scheme has failed to expand coverage to the poor, vulnerable and informal sector populations.^51^ Private voluntary health insurance (VHI) has shown poor potential to extend health insurance coverage.^52^ Exemption schemes and waivers targeted at the poor and vulnerable groups have not been effective in increasing enrolment and addressing the barriers to accessing healthcare for these groups due to problems associated with targeting. The poor, vulnerable and informal sector populations are disproportionately exposed to catastrophic and impoverishing effects of high OOP payments. NHIS as an agency of government operate under the Federal Ministry of Health (FMoH).^43^ This agency registers and accredits health maintenance organizations (HMOs) and healthcare providers (HCPs). HMOs collect contributions and pay monthly capitation or fee-for-service (FFS) to HCPs for services provided. HCPs are registered private and public hospitals and clinics at the primary, secondary and tertiary levels that provide healthcare services to NHIS enrolees who are registered through an HMO.^43^ Despite the disentanglement of the NHIS (as an implementing and regulatory agency), HMOs and HCPs; the NHIS has been bedevilled with poor governance, mismanagement of fund, corruption as well as lack of transparency and accountability. NHIS fund is obtained from general government revenues; premium contributions; returns from investments as well as grants or donations. The NHIS covers over 95% of disease conditions that affect the Nigerian population. The benefit package under the FSSHIP which serve most of the NHIS enrolees includes out-patient services, in-patient services, maternity care for up to four live births, emergencies, preventive care including immunisation, consultation with specialists, eye examination and care, preventive dental care and pain relief as well as a range of prostheses.^43^ Some of the benefits the NHIS has brought since its inception is access to healthcare for formal sector workers and their dependents as well as tertiary level students who pay a premium of N2000 (US$5) per annum. The NHIS has also reduced OOP health payments for this group of enrolees.

## Methods

### Data Source

Secondary data from the 2013 Nigeria Demographic and Health Survey (NDHS) were used for the study. NDHS is a nationally representative cross-sectional study conducted by the National Population Commission (NPC) with funding by United States Agency for International Development (USAID), the United Kingdom Department for International Development (DFID) through Partnership for Transforming Health Systems Phase II (PATHS2), and the United Nations Population Fund (UNFPA) with technical support from ICF International.^53^ The NDHS 2013 provides updated estimates of the basic demographic and health indicators covered in the earlier surveys such as fertility levels, marriage, fertility preferences, awareness and use of family planning methods, child feeding practices, nutritional status of women and children, adult and childhood mortality, awareness and attitudes regarding HIV/AIDS, in addition, to information on violence against women.^53^

### Study Design

This was a retrospective cross-sectional study comprised of 38 948 women aged 15-49 years.

### Data collection

Relevant data for the study were extracted from the 2013 NDHS dataset. Among the numerous variables in the women’s recode, only eleven variables were selected for the purpose of this study. The data were thereafter cleaned, re-categorized and recoded as necessary.

### Variables Measurement

#### Dependent Variable

The dependent and/or outcome variable is health insurance coverage coded as 1 if covered by health insurance and 0 otherwise.

#### Independent Variables

The independent and/or explanatory variables were guided by Andersen’s Behavioural Model,^54^ Grossman’s Model of Demand for Health^55^ and literature review on the determinants of enrolment in NHIS.^3,21-42^ In Andersen’s Behavioural Model, the variables that determine women’s decision to enrol in the NHIS are categorized into three namely: predisposing factors (age, gender, ethnicity and household head characteristics); enabling factors (location, geo-political zone, education, health insurance status and household income); and need factors (perceived severity of illness, self-reported health status, presence of physician diagnosing chronic diseases and overweight). These factors interact with each other to determine whether or not women enrol in the NHIS. According to Grossman, the demand for health is influenced by factors such as age, wage rate and education.^55^ Grossman’s model of demand for health proposes that health can be viewed as a durable capital stock that produces an output of healthy time.^55^ A person determines his optimal stock of health capital at any age by equating the marginal efficiency of this capital to its user cost in terms of the price of gross investment.^55^ The model explains variations in both health medical care among persons in terms of variation in supply and demand curves for health capital.^55^ Grossman’s model predicts that if the rate of depreciation increase with age, at least after some point in the life cycle, then the quantity of health capital demanded would decline over the life cycle.^55^ Another prediction is that a consumer’s demand for health and medical care should be positively correlated with his wage rate.^55^ Lastly, Grossman’s model predicts that if education increases the efficiency with which gross investments in health are produced, then the more educated would demand a larger optimal stock of health.^55^ Grossman’s model of demand for health enables one to study the effects of demographic variables like age and education without assuming that these variables are positively or negatively correlated with consumers’ “tastes’’ for health.^55^

In our study, age refers to the age of women at the time of the household survey and was categorized as 15-24, 25-34, and 35+ years. Educational status refers to the highest level of education attained by women and was categorized into no education, primary education, secondary or higher education. The place of residence refers to the location of a woman’s residence, grouped into urban and rural areas. Nigeria is divided into six geo-political zones: North Central, North East, North West, South East, South West and South South. A SES index was constructed using principal components analysis (PCA) based on data from variables on household ownership of assets and housing conditions.^56^ These variables include ownership of a car/truck, ownership of radio, ownership of refrigerator, ownership of bicycle, ownership of motorcycle, main wall material, main floor material, main roof material, type of fuel for cooking, source of electricity, source of drinking water, time to get to water source and type of toilet facility used. PCA generated factor score on each household asset. The resulting asset scores were standardised while the standardised scores were used to generate SES quintile as poorest, poorer, middle, richer and richest. Marital status describes the type of marital relationship a woman was in and was categorised as never married, married, living with partner, widowed, divorced and separated. Religion refers to the religious affiliation of women and was recoded as Christian, Muslim and Traditionalist/others. Employment status describes the type of employment women were engaged in and was recoded as not working, formal and informal worker. Household size was defined according to the survey questionnaire and recoded as less than five members and five or more members. Gender of household head was defined according to the survey questionnaire and recorded as male and female. Table 1 presents a summary of the study variables.

**Table 1:** Summary of the Study Variables

**Variables**	**Definition of Variables**	**Variables Description/Coding**
Health insurance coverage	Coverage by any form of health insurance	Dummy: 1 = Yes, 0 = No
Age	Age of women at the time of the household survey	Ordered categorical variable with three outcomes 0-15-24 years 1-25-34 years 2-35 years or older
Educational status	The highest level of education attained by women	Ordered categorical variable with three outcomes 0- No education 1- Primary education 2- Secondary or higher education
Place of residence	Location of a woman’s residence	Dummy: Rural = 1, Urban = 0
Geo-political zone	Region where respondents reside	Categorical variable with six outcomes 1- North Central 2- North East 3- North West 4- Soouth East 5- South South 6- South West
Socio-economic status	Socio-economic position of women	Ordered categorical variable with five outcomes 1- Poorest 2- Second poorest 3- Middle 4- Second richest 5- Richest
Marital status	The type of marital relationship a woman was in	Ordered categorical variable with six outcomes 0- Never married 1- Married 2- Living with partner 3- Widowed 4- Divorced 5- Separated
Religion	Religious affiliation of women	Ordered categorical variable with three outcomes 0- Christian 1- Muslim 2- Traditionalist/others
Employment status	The type of employment women were engaged in	Ordered categorical variable with three outcomes 0- Not working 1- Formal worker 2- Informal worker
Household size	The number of household members	0- Less than five members 1- Five or more members
Gender of household head	Gender of household head	Dummy: 1 = Female, 0 = Male

### Statistical Analysis

Data were analysed using STATA version 12 software. Descriptive statistics was used to analyse the household and individual characteristics of the study sample as well as the outcome variable in the form of frequency tables and simple percentages. Pearson’s chi-square analysis was used to test for associations between independent variables and health insurance status. Thereafter, multivariate logistic regression was used to examine associations between the dependent variable (health insurance coverage) and the independent variables (age, educational status, place of residence, geo-political zone, SES, marital status, religion, employment status, household size and gender of household head). Binary logistic regression was run since the dependent variable (health insurance coverage) has binary outcomes (*Yes or No*). Using the equation in the binary logistic regression model:

y = *β*_o_ + *β*_i_*X*_i_ + *β*_2_*X*_2_ + *β*_3_*X*_3_.....*β*_n_*X*_n_ + *E*_i_ (1)

Where y is the outcome/dependent variables, *β*_o_ is the constant/intercept, *β*_i_…*β*_n_ are the regression coefficients and the *X*_i_….*X*_n_ are a collection of independent/explanatory variables and *E*_i_ is the error term. Tests were done at a confidence level of 95% and at 5% significance level.

## Results

### Descriptive Statistics

Figure shows health insurance coverage for women of reproductive age in Nigeria. Only 2.1% of women had health insurance coverage. A total of 97.9% did not have health insurance coverage.

**Figure:**
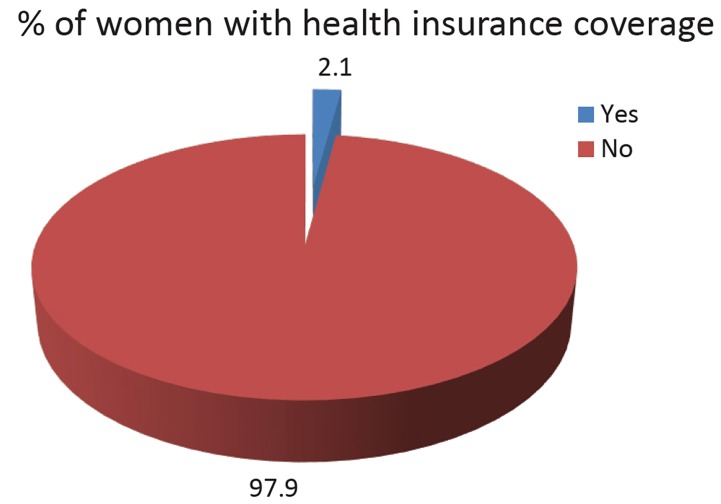
Health Insurance Coverage for Women of Reproductive Age in Nigeria.

### Predictors of NHIS Enrolment Among Women of Reproductive Age

Table 2 presents the study population characteristics and bivariate analysis of the predictors of NHIS enrolment among women of reproductive age.

**Table 2:** Study Population Characteristics and Bivariate Analysis of the Predictors of NHIS Enrolment Among Women of Reproductive Age

**Demographic and Socio-economic Characteristics**	**Health Insurance Coverage**			***P *** **Value**
	**Total** (**N= 38 948)** **No. %**	**No (n = 38 128)** **No. %**	**Yes (n = 820)** **No. %**	
Age				<.001**
15-24	14** **619 (37.50)	14** **423 (98.66)	196 (1.34)	
25-34	12** **410 (31.90)	12** **099 (97.49)	311 (2.51)	
35 +	11** **919 (30.60)	11** **606 (97.37)	313 (2.63)	
Educational status				<.001**
No education	13** **740 (35.30)	13** **704 (99.74)	36 (0.26)	
Primary education	7104 (18.20)	7051 (99.25)	53 (0.75)	
Secondary or higher education	18** **104 (46.50)	17** **373 (95.96)	731 (4.04)	
Place of residence				<.001**
Urban	15** **545 (39.90)	14** **945 (96.14)	600 (3.86)	
Rural	23** **403 (60.10)	23** **183 (99.06)	220 (0.94)	
Geo-political zones				<.001**
North Central	6251 (16.00)	6001 (96.00)	250 (4.00)	
North East	6630 (17.00)	6523 (98.39)	107 (1.61)	
North West	9673 (24.80)	9613 (99.38)	60 (0.62)	
South East	4462 (11.50)	4372 (97.98)	90 (2.02)	
South South	6058 (15.60)	5876 (97.00)	182 (3.00)	
South West	5874 (15.10)	5743 (97.77)	131 (2.23)	
Socio-economic status				<.001**
Poorest	6602 (17.00)	6599 (99.95)	3 (0.05)	
Poorer	7515 (19.30)	7504 (99.85)	11 (0.15)	
Middle	8001 (20.50)	7937 (99.20)	64 (0.80)	
Richer	8450 (21.70)	8285 (98.05)	165 (1.95)	
Richest	8380 (21.50)	7803 (93.11)	577 (6.89)	
Employment status				<.001**
Not working	14** **262 (36.60)	14** **034 (98.40)	228 (1.60)	
Formal worker	2162 (5.60)	1878 (86.86)	284 (13.14)	
Informal worker	22** **524 (57.80)	22** **216 (98.63)	308 (1.37)	
Religion				<.001**
Christian	19** **838 (50.90)	19** **245 (97.01)	593 (2.99)	
Muslim	18** **578 (47.70)	18** **353 (98.79)	225 (1.21)	
Traditionalists/others	532 (1.40)	530 (99.62)	2 (0.38)	
Marital status				.537
Not married	9820 (25.20)	9617 (97.93)	203 (2.07)	
Married	26** **403 (67.80)	25** **836 (97.85)	567 (2.15)	
Living with partner	871 (2.20)	853 (97.93)	18 (2.07)	
Widowed	993 (2.50)	971 (97.78)	22 (2.22)	
Divorced	432 (1.10)	427 (98.84)	5 (1.16)	
Separated	429 (1.10)	424 (98.83)	5 (1.17)	
Household size				<.001**
Less than five members	12** **829 (32.90)	12** **507 (97.49)	322 (2.51)	
Five or more members	26** **119 (67.10)	25** **621 (98.09)	498 (1.91)	
Gender of household head				<.001**
Male	31** **838 (81.70)	31** **209 (98.02)	629 (1.98)	
Female	7110 (18.30)	6919 (97.31)	191 (2.69)	

Abbreviation: NHIS, National Health Insurance Scheme; SES, Socio-economic status. ** *P* < 0.05.

Results from the bivariate analysis of the association between health insurance coverage and explanatory variables showed that the percentage of the insured was significantly higher among women aged 35 years or older (2.63%). The proportion of the insured was significantly higher among women with secondary or higher education (4.04%). The percentage of the insured was significantly higher among women residing in urban areas (3.86%). The proportion of the insured was significantly higher among women from North Central region (4.00%) and South South region (3.00%). The percentage of the insured was higher among women from richest households (6.89%). The proportion of the insured was significantly higher among women who are formal worker (13.14%). The percentage of the insured was significantly higher among women who are Christians (2.99%). The proportion of the insured was significantly higher among women with less than five members in their household (2.51%). The percentage of the insured was significantly higher among women from households with female heads (2.69%). Only variables that were statistically significant in the bivariate analysis were included in the logistic regression model.

Table 3 presents results of the predictors of enrolment in the NHIS among women of reproductive age using logistic regression model.

**Table 3:** Predictors of NHIS Enrolment Among Women of Reproductive Age Using Logistic Regression Model

**Demographic and Socio-Economic Characteristics**	**Health Insurance Coverage**	
	**OR**	**95% CI**
Age		
15-24	1	1
25-34	1.71**	(1.39-2.09)
35 +	2.23**	(1.79-2.78)
Educational status		
No education	1	1
Primary education	1.24	(0.79-1.93)
Secondary or higher education	3.07**	(2.08-4.52)
Place of residence		
Urban	1	1
Rural	0.86	(0.72-1.04)
Geo-political zone		
North Central	1	1
North East	1.04	(0.81-1.34)
North West	0.52**	(0.38-0.70)
South East	0.36**	(0.28-0.47)
South South	0.57**	(0.46-0.70)
South West	0.27**	(0.21-0.34)
SES		
Poorest	1	1
Poorer	2.62	(0.73-9.47)
Middle	10.77**	(3.30-35.13)
Richer	21.43**	(6.60-69.64)
Richest	57.67**	(17.66-188.30)
Employment status		
Not working	1	1
Formal worker	2.82**	(2.27-3.50)
Informal worker	0.83	(0.68-1.01)
Religion		
Christian	1	1
Muslim	0.90	(0.74-1.10)
Traditionalists/others	0.34	(0.08-1.40)
Household size		
Less than five members	1	1
Five or more members	1.09	(0.93-1.27)
Gender of household head		
Male	1	1
Female	1.02	(0.85-1.22)

Abbreviations: OR, odds ratio; NHIS, National Health Insurance Scheme; SES, Socio-economic status. ** *P* < 0.05.

Results showed that the odds of having health insurance coverage was significantly higher for women aged 25-34 years (odds ratio [OR]: 1.71; 95% CI: 1.39-2.09) and 35 years or older (OR: 2.23; 95% CI: 1.79-2.78) compared with women who are aged 15-24 years. Women with secondary or higher education were 3.07 times more likely to have health insurance coverage compared to women with primary education and no education. The odds of having health insurance coverage was significantly lower among women from North West region (OR: 0.52; 95% CI: 0.38-0.70), South East region (OR: 0.36; 95% CI: 0.28-0.47), South South region (OR: 0.57; 95% CI: 0.46-0.70) and South West region (OR: 0.27; 95% CI: 0.21-0.34). Women from middle income, richer and richest households were 10.77, 21.43 and 57.67 times more likely to have health insurance coverage compared with women from poorer and poorest households. The odds of having health insurance coverage was significantly higher among women employed in the formal sector (OR: 2.82; 95% CI: 2.27-3.50) compared with women not working and women working in the informal sector.

## Discussion

This study examined the predictors of enrolment in the NHIS among women of reproductive age in Nigeria. We found that a high proportion of women (97.9%) had no health insurance coverage. This could be due to large proportion of the Nigerian population in the informal sector, high level of poverty and voluntary contributory health insurance scheme targeted at the poor and informal sector workers. However, the low level of enrolment in the NHIS is consistent with other studies in Africa.^3,30,31,35^ Our study also revealed that women aged 25-34 years and 35 years or older were more likely to have health insurance coverage. This suggests that older women have financial security because they are usually employed whether in the formal or informal sector compared with younger ones. Also, older women have high risk of illness due to increase in age and this may lead to their increased investments in health including the purchase of health insurance in order to protect themselves from financial uncertainties and the risk of illness.^22,27,31,35,57^ However, the finding corroborates results from similar studies.^3,22,25,26,27,31,35^ Results showed that women with secondary or higher education were more likely to have health insurance coverage compared with women with no education. A possible reason is that women who are educated have more knowledge about the advantages of health insurance and make informed choices about their health including purchasing health insurance. This finding is supported by results from other studies.^3,22,23,26,31,33,35,36,39,40^ We also found that place of residence was not a significant predictor of enrolment in the NHIS among women. A possible explanation is that women from poor households who are unable to pay insurance contributions live in both rural and urban areas. Also, women who work in the informal sector are also spread across rural and urban areas. However, this finding is in contrast to results from other studies.^22,26,27,34,35,38^ In this study, women in North West region, South East region, South South region and South West region were less likely to have health insurance. The low level of enrolment in these geographical locations could be due to the fact that they are predominantly rural and have a high proportion of women from poor households who do not have knowledge of the advantages of health insurance. Furthermore, most of the health insurance organizations are concentrated in urban areas and not within the reach of women residing in rural areas. This finding is consistent with similar studies that found that geopolitical zone was associated with lower odds of having health insurance coverage.^31,35^ Also, women from middle income, richer and richest households were more likely to have health insurance coverage compared with women from poorest households. A possible explanation is that women in poorest households have limited access to resources and cannot afford health insurance plans and experience difficulty in paying premium contributions. The finding is consistent with other studies that showed that higher SES is an important predictor of enrolment in NHIS.^3,22,23,26,31,33,34,37,38,41,42^ Being a formal worker was a significant predictor of enrolment in NHIS among women of reproductive age in this study. A plausible explanation for this observation is that formal workers earn income that is easily deducted as part of contribution towards the purchase of health insurance. Also, this could be due to the fact that the unemployed and workers in the informal sector are not economically empowered to pay premium contributions. This finding is consistent with similar studies in Africa.^3,22,24,31,33,35,40^ The wide gap between health insurance coverage for formal and informal workers have implications on the use of voluntary contributory health insurance schemes to expand health insurance coverage to informal sector workers. Both the VHI and CBHI schemes have provided limited coverage to the poor and informal sector workers. Evidence suggests that it is difficult for contributory insurance schemes to achieve UHC given a large informal sector population.^58-60^ This means that the current health financing mechanism cannot assist the country in moving towards UHC. Results from this study also showed that religious affiliation was not a significant predictor of enrolment in the NHIS among women. A possible explanation is that enrolment in the NHIS is not opposed by religious institutions due to the advantages of health insurance. This is in line with findings from a similar study in Ghana.^34^ In this study, household size was not a significant predictor of enrolment in the NHIS among women in Nigeria. This suggests that all members of a household regardless of their size should be covered by health insurance. However, the finding is in contrast to similar studies.^3,22,26,27^ Gender of household head was not a significant predictor of the NHIS enrolment among women. A plausible explanation is that both male and female headed households face the risk of ill-health and should be concerned about protecting their household. A study in rural Burkina Faso supports this finding^23^ but the result is in contrast to similar studies in Africa that found that women residing in female-headed households were more likely to have health insurance coverage compared with women from male-headed households.^27,31^

Findings from this study have implications for policy-makers in Nigeria. First, some factors predict enrolment in the NHIS among women of reproductive age. Policy-makers need to target these factors in their effort to increase the level of enrolment in the NHIS among women. Second, women from poor households and those working in the informal sector are excluded from the NHIS. This implies that the NHIS is not a pro-poor health financing policy. Therefore, there is a need to adopt a tax-financed non-contributory health insurance scheme as the primary financing mechanism in order to expand health insurance coverage to women from poor households and those working in the informal sector. The provision of health insurance to poor women and those working in the informal sector represents a bottom-up approach to expanding health insurances coverage which is a viable option for developing countries including Nigeria.^61^

## Limitations of the study

Our study has some limitations that nevertheless do not invalidate our work. First, the study used cross-sectional secondary data; hence, causality for the factors associated with enrolment in the NHIS could not be established. Second, findings from our study are affected by recall bias due to self-reported information. Third, the study did not include important variables such as health status, type of facility visited and type of illness suffered due to their unavailability in the NDHS dataset. Finally, data from the 2013 NDHS may be old in informing policy decisions but it is the most recent NDHS dataset available to the public after the release of the 2018 NDHS data.

## Conclusion

This study concludes that health insurance coverage among women of reproductive age in Nigeria is very low. Additionally, socio-demographic factors such as age, education, geo-political zone, SES and employment status were significant predictors of enrolment in NHIS among women of reproductive age. This implies that the NHIS is not a pro-poor health financing policy as a high proportion of women from the poorest and poorer households lack health insurance coverage. Furthermore, effort to expand health insurance coverage to women from poor households and those working in the informal sector through voluntary contributory health insurance schemes may not be feasible due to financial constraint and difficulty in collecting contributions among these groups. Therefore, governments and policy-makers should establish a tax-based health financing mechanism specifically targeted at women who are young, uneducated, from poorest households, unemployed and working in the informal sector. Extending health insurance coverage to women from poor households and those who work in the informal sector through a tax-financed non-contributory health insurance scheme would accelerate progress towards UHC.

## Ethical issues

In obtaining the micro data, a request was made on the DHS program website on March 6, 2018 and approval was granted to download the data on the same day, hence, there were no ethical issues of concern. Issues of informed consent, confidentiality, anonymity and privacy of the study sample were addressed by the Institutional Review Board (IRB) of ICF Macro International in the United States and National Health Research Ethics Committee (NHREC) of the Federal Ministry of Health (FMoH).

## Competing interests

Authors declare that they have no competing interests.

## Authors’ contributions

BSA: Conception and design; acquisition of data; analysis and interpretation of data; and drafting of the manuscript. SMK: Analysis and interpretation of data; and drafting of the manuscript. Both authors reviewed the manuscript for important intellectual content and approved the final draft for publication.

## Authors’ affiliations

^1^Department of Community Health & Primary Care, College of Medicine, University of Lagos, Lagos, Nigeria. ^2^Department of Public Health Sciences, Karolinska Institutet, Stockholm, Sweden.

## Key messages

**Implications for policy makers**

- The National Health Insurance Scheme (NHIS) has not provided access to healthcare services and financial risk protection for a large proportion of women and poor households in Nigeria.
- Voluntary contributory health insurance scheme for women from poor households and those working in the informal sector may not be feasible in expanding health insurance coverage due to financial constraint and difficulty in collecting contributions among these groups.
- Addressing the problem of poor uptake of health insurance among women from poor households require a special protection and tax-based health financing mechanism.

**Implications for the public**

There are concerns about the effectiveness of the National Health Insurance Scheme (NHIS) to expand health insurance coverage to women from poor households and those working in the informal sector. The law establishing the NHIS need to be re-examined while the expansion of health insurance coverage for women of reproductive age who remain uninsured must be considered. Governments and policy-makers need to engage women of reproductive age in discussing feasible pathways for achieving universal health coverage (UHC) as a goal of the Sustainable Development Goals (SDGs) by 2030. Despite the goal of the NHIS to ensure that all population groups regardless of their socio-economic status (SES) have access to quality and affordable healthcare services, majority of women of reproductive age are not enrolled in the scheme. However, there is a need for women of reproductive age to demand health financing reform and political leadership from governments and policy-makers.
